# Loneliness as a mediator between family separation and life satisfaction among Ukrainian refugee women in Germany

**DOI:** 10.1038/s41598-026-55664-w

**Published:** 2026-05-30

**Authors:** Nataliia Levchuk, Domantas Jasilionis, Lisa Kriechel, Martin Bujard

**Affiliations:** 1 Max Planck Institute for Demographic Research (MPIDR), Rostock, Germany; 2 Federal Institute for Population Research (BIB), Wiesbaden, Germany; 3 Mykhailo Ptukha Institute for Demography and Life Quality Research, Kyiv, Ukraine; 4 Max Planck - University of Helsinki Center for Social Inequalities in Population Health (MaxHel Center), Rostock, Germany; 5 Institute of Medical Psychology, Medical Faculty, University Heidelberg, Heidelberg, Germany

**Keywords:** Geography, Geography, Health humanities, Psychology, Psychology, Sociology

## Abstract

The Russian invasion of Ukraine has led to one of the largest population displacements in Europe since World War II. Different from other recent refugee flows, Ukrainian refugees are predominantly women, often separated from partners who remained behind. Using data from the “IAB-BiB/FReDA-BAMF-SOEP Survey on Refugees from Ukraine in Germany,” this cross-sectional study examines how separation-related loneliness and integration barriers are associated with life satisfaction among displaced women who arrived in Germany in 2022. Structural equation modeling showed that the association between forced family separation and life satisfaction operated entirely through indirect pathways involving loneliness and integration barriers. Loneliness accounted for the largest share of this indirect association and acted as a key intermediary, being linked to both lower life satisfaction and greater integration challenges, including economic and health concerns and reduced social integration. These results suggest that loneliness may be an important emotional pathway connecting separation to broader integration difficulties and lower well-being, particularly in the early stage of resettlement. Timely measures to address loneliness could help support integration and improve well-being of refugee women affected by separation.

**Supplementary Information:**

The online version contains supplementary material available at 10.1038/s41598-026-55664-w.

## Introduction

War-related displacement has profound and far-reaching consequences for various dimensions of individuals’ lives. As a life-disrupting experience, forced displacement significantly impacts the psychological well-being and health of refugees, not only due to the traumatic events endured in their home countries but also because of the challenges associated with adapting to new cultural, linguistic, and social environments^1–3^. Among the many difficulties they encounter, family separation and loneliness are particularly common and impactful^4^. Separation has been linked to heightened psychological distress and lower life satisfaction, especially among those who wish to reunite with their families^5–9^. Some studies also suggest that fear for safety of family members remaining in the country of origin can inhibit refugees’ ability to function daily or make social connections in the host country^7^. Compared to migrants and host population*s*, refugees report higher levels of loneliness^10^, which further contributes to their mental health challenges. Increased loneliness during resettlement is associated with poorer general health, lower life satisfaction, and reduced health-related quality of life^11,12^.

The Russian invasion of Ukraine in February 2022 and the subsequent prolonged war has caused an unprecedented wave of migration to Europe, with Germany receiving the largest number of Ukrainian refugees— 1.21 million as of April 2026^13^. Compared to other refugee groups in Europe, Ukrainian refugees show quite distinct features. First, this group is predominantly composed of women, with half accompanied by minors^14^. Second, a substantial proportion of these women are married but currently residing in Germany while separated from their partners in Ukraine^15^. Due to martial law, nearly all men of conscription age (with a few exceptions) are required to remain in the country and cannot travel abroad. Thus, understanding the determinants of well-being for this distinctive group is crucial for their successful integration into German society.

Prior research shows that loneliness can have various causes, with partner separation being one of its strongest predictors^16–18^. Women are especially vulnerable to emotional loneliness following the loss or absence of a partner due to separation^19,20^. In the context of Ukrainian refugee women, the forced and prolonged separation from their partners due to the war likely increases their risk of experiencing loneliness and related distress. However, most prior studies on refugee wellbeing in Germany have focused on forcibly displaced populations from Syria, Afghanistan, and Iraq, which usually rely on male-dominated samples^2,9,12,21–23^.

Despite growing interest in the well-being of war-displaced Ukrainians, much of the recent research has focused on general psychological outcomes^24–26^. For example, studies from Germany indicate that Ukrainian refugees reported lower life satisfaction compared to the general population^14^ , with women particularly vulnerable to poorer mental health^27^. Other research confirm that Ukrainian refugees are more prone to depression and psychological distress^28,29^. However, loneliness as a specific consequence of war-related partner separation remains understudied and is often addressed only within the broader context of mental health issues^30–32^.

In addition to loneliness, refugees may face a range of various post-arrival challenges that can also adversely affect their well-being, including language difficulties, social isolation, discrimination, and economic hardship^2,33^. For instance, language barriers and social connections with locals are crucial for integration and are associated with higher life satisfaction^2,34^ and improved mental health. Refugees with lower social support exhibit higher distress levels, as indicated by biomarker studies^35^. Another major integration barrier is perceived discrimination and a lack of welcome in the host country: refugees who feel unwelcome report lower life satisfaction^36^. Economic and financial challenges also play a critical role, as financial stability is strongly linked to higher life satisfaction and can buffer the negative effects of forced displacement and separation^37^. Additionally, health concerns hinder integration, with better subjective health status being associated with greater life satisfaction^34^.

However, the relationship between these post-migration factors and loneliness might be complex. On the one hand numerous studies have shown that social isolation and a lack of integration increase feelings of loneliness^10,18,38^. On the other hand, some evidence suggests that loneliness itself can hinder active integration, thereby contributing to deteriorating mental health^39,40^. Furthermore, prior research has rarely examined these factors together or tested potential pathways linking family separation, loneliness, integration difficulties, and subjective well-being. To our knowledge, no study systematically explored the interplay between these factors and their combined effects on the life satisfaction of Ukrainian refugees.

This cross-sectional study addresses two research gaps. First, it focuses on perceived loneliness resulting from war-related family separation, a distinct and underexplored form of distress among women who were recently forcibly displaced. Second, it examines how loneliness and integration barriers jointly relate to life satisfaction using structural equation modeling, contributing to understanding of the pathways linking separation, loneliness, and well-being.

Our main research question is: In the early stage of resettlement, how is loneliness arising from forced family separation due to war-related displacement associated with integration barriers and life satisfaction among refugee women?

Specifically, we test the following hypotheses:

### **H1**

*separation is negatively associated with life satisfaction*

### **H2**

*loneliness mediates this relationship:*

### **H3**

*loneliness acts as a key intermediary linking forced family separation to greater integration barriers, which are in turn associated with lower life satisfaction.*

Using a unified analytical framework and a structural equation model with a serial-parallel mediation design, we analyze the multidimensional role of loneliness in relation to the life satisfaction of partnered displaced women-those who are married or in stable relationship. By testing these pathways, our study offers new insights on how separation-related loneliness and early-stage integration difficulties are associated with women’s well-being in the context of displacement.

## Methods

### Participants

The study is based on a secondary data analysis of the first wave of the IAB-BiB/FReDA-BAMF-SOEP Survey, “Refugees from Ukraine in Germany”, conducted between August and September 2022, shortly after the escalation of the war in Ukraine^14,41^. The survey was organized by the Institute for Employment Research (IAB) of the Federal Employment Agency, the Federal Institute for Population Research (BiB), the Family Research and Demographic Analysis (FReDA) project, the Research Center of the Federal Office for Migration and Refugees (BAMF-FZ) and the Socio-Economic Panel (SOEP) at DIW Berlin. Data collection was carried out by the infas Institute for Applied Social Science.

Respondents were randomly selected through a two-stage sampling process: first, 100 locations (cities and counties) across the 16 federal states in Germany were chosen based on the distribution of Ukrainian refugees in the Central Register of Foreigners (“Ausländerzentralregister”) and then individuals were sampled from the population register (“Einwohnermelderegister”). The survey sample is considered representative of Ukrainians who fled to Germany during the field period. Data were collected using a mixed-mode push-to-web design, combining online and postal questionnaires^42^. The response rate was 25%, which is comparable to recruiting waves of studies with similar research design^43^.

A total of 11,754 Ukrainians aged 18–87 participated in the survey (*M*_*age*_ = 41.15, *SD*_*age*_ = 13.63, 81.5% female). We excluded 204 respondents who arrived before February 24, 2022, and 17 cases with diverse or unknown sex. Our subsample includes women in partnership, defined as those who are married or in a stable relationship (N = 5,488, *M*_*age*_ = 40.59, *SD*_*age*_ = 11.71, age range: 18–71). After excluding 177 cases (3.2%) with partners who remained in a third country (other than Germany and Ukraine), the final analytical sample consists of 5,311 women aged 18 to 71 (*M*_*age*_ = 40.7, *SD*_*age*_ = 13.4). Of these, 3087 (58.6%, *M*_*age*_ = 40.1, *SD*_*age*_ = 10.2) had partners who remained in Ukraine, while 2197 (41.4%; *M*_*age*_ = 40.1, *SD*_*age*_ = 13.4) had partners who stayed in Germany (with 27 cases (0.5%) missing). The proportion of missing values across variables is generally low, ranging from 0.11% (age) to 1.26% (health concerns), and a total of 292 cases (5.5%) have at least one missing value.

Most participants arrived in Germany in March 2022, shortly after the start of the full-scale invasion. The average month of arrival was the same for separated and non-separated women, with no statistically significant difference between the groups (*p* = 0.344), despite a small variation in the overall distribution (*p* < 0.001). Notably, 94% of the women arrived within the first four months of the war, representing refugees in the early stages of resettlement.

Throughout this manuscript, we use the terms “Ukrainian refugee women” and “displaced Ukrainian women” interchangeably. Although Ukrainians in Germany are granted residence under the EU’s Temporary Protection Directive (Directive 2001/55/EC) rather than formal refugee status through the asylum system, both terms are widely used in academic literature to refer to those who fled Ukraine due to the war.

### Measures

#### Dependent variable

Life satisfaction is a widely used subjective measure of overall well-being, reflecting individuals’ perceptions and assessments of their life situations, achievements, and overall happiness^44^. It was measured using a single-item global life satisfaction Likert scale based on the question: “How satisfied are you with your life in general?”, with responses ranging from 0 (“Totally dissatisfied”) and 10 (“Totally satisfied”).

#### Independent variable

Family separation was determined based on a question about the current place of residence of respondent’s partner: “In what country does your partner live now?”. Women were categorized as separated if their partners lived in Ukraine and non-separated if their partners lived in Germany. This definition focuses exclusively on separation from a partner and does not account for other forms of separation that may occur due to war-related displacement, such as separation from children or other close family members.

### Mediators

Loneliness. It was assessed using a direct, single-item measure in which participants were asked to what extent the statement “I feel lonely” applied to them. Responses were recorded on a 5-point scale ranging from 1 (“Does not apply at all”) to 5 (“Fully applies”). For analysis, we rescaled this variable to range from 0 to 4. Although this measure does not explicitly differentiate between social and emotional loneliness, it captures participants’ subjective experience of loneliness at the time of data collection, which occurred shortly after displacement and separation from their partners.

Integration barriers. They represent subjective challenges that refugees may encounter when adapting to a host country. Instead of relying on objective indicators such as employment status, we assessed perceived barriers in three key areas: language proficiency, social integration, and economic/health concerns. As further explained below, they are identified through exploratory and confirmatory factor analyses. The original variables are measured as follows. Language proficiency is assessed through three self-reported items: “How well do you a) speak, b) write, and c) read German?” with response options ranging from 1 (“Very good”) to 5 (“Not at all”). Social integration is measured with the question: “How often do you spend time with a) Germans, b) people from Ukraine who are not your relatives?” rated from 1 (“Every day”) to 6 (“Never”). Feeling welcome in Germany is determined by asking, “Did you have a feeling after arriving in Germany that you are welcome?” with answers from 1 (“Absolutely yes”) to 5 (“Not at all”). Economic and health concerns are measured with the question: “Are you worried about a) your economic situation, b) your health?” with options from 1 (“Serious concern”) to 3 (No problem”). Perceived financial difficulties are assessed with the question: “When you think about your current income and the money available to you and any family members who fled with you, how are you coping?” rated from 1 (“With great difficulties”) to 6 (“Very good”). Since the original scales of the variables representing possible integration barriers vary all items were reversed so that higher values consistently reflected greater levels of the respective construct.

### Control variables

Control variables included: age (continuous), education (high tertiary education vs. lower education), employment status (employed full-time/part-time vs. not employed/in education), accommodation type (private vs. non-private), having children (yes/no), and intention to stay in Germany (planning to stay for several years or permanently vs. planning to stay until the end of the war, for a maximum one year, or not sure).

### Statistical analysis

After examining correlations between the study variables, we employed structural equation modeling (SEM) with mediation analysis to assess the role of loneliness arising from forced family separation due to war-related displacement in the associations between separation, integration barriers, and life satisfaction among refugee women. The analysis proceeded as follows.

First, we estimated a direct association model (Model 1) to assess the relationship between family separation and life satisfaction: Family separation → Loneliness. Given the context of forced displacement, we expect that the absence of a partner is linked to lower life satisfaction among women.

In Model 2, we introduce loneliness as a single mediator to examine its indirect association with life satisfaction: Family separation → Loneliness → Life Satisfaction. As a subjective emotional response to separation from a partner, loneliness is expected to mediate the relationship by capturing the emotional costs of separation.

Models 3 examined more complex pathways involving both loneliness and integration barriers as potential mediators. We hypothesized that forced partner separation is associated with increased emotional loneliness, which may be linked to greater integration difficulties and, in turn, lower life satisfaction. Specifically, Model 3 tested a serial-parallel mediation model, in which loneliness functions as a serial mediator and three integration barrier factors operate as parallel mediators. Accordingly, our model assumes the following directional pathways:

Family Separation → Loneliness → Integration barrier factors → Life Satisfaction.

Loneliness → Life Satisfaction (direct parallel path).

Family Separation → Life Satisfaction (direct path).

Integration barriers. To identify which aspects of integration difficulties are most relevant to displaced women’s experiences, we first conducted an exploratory factor analysis to uncover the underlying dimensions of the early-stage integration difficulties and determine the optimal number of factors. This was followed by a confirmatory factor analysis to validate the factor structure and estimate factor loadings.

To assess the suitability of these data for factor analysis, we used the Kaiser–Meyer–Olkin index and Bartlett’s test. A KMO value above 0.6 is considered acceptable for demonstrating factorial simplicity^45^. For comparability and consistency in interpretation, the variables representing possible integration barriers were first transformed using z-standardization. Although the transformation expresses values in standard deviation units, the undelaying relationship and relative differences among the variables are preserved.

The results of Mardia’s Test for multivariate normality indicate that the data do not follow multivariate normal distribution, as evidenced by both the skewness statistic (6231.19, *p* < 0.001) and the kurtosis statistic (37.07; *p* < 0.001). To address this violation, the Minimum Residual (MinRes) method was chosen for the EFA as this method is robust to non-normality. It relies on correlations rather than raw values, making it an appropriate choice for the current analysis. EFA was conducted on the z-standardized integration-barrier-related variables with oblique (Oblimin) rotation, allowing for correlations between factors. Factors were retained based on standard criteria, including the scree test, eigenvalues greater than 1^46^ , with standardized factor loadings greater than 0.4^47^, though lower thresholds were considered acceptable for exploratory constructs. We then conducted CFA to confirm the factor structure identified in EFA. Model fit was assessed using standard fit indices, including the comparative fit index (CFI), Tucker-Lewis index (TLI), root mean square error of approximation (RMSEA), and standardized root mean square residual (SRMR). CFI and TLI values above 0.95 were considered indicative of good fit, while RMSEA values below 0.08 were deemed acceptable^48^. SRMR values were expected to be below 0.08^49^. Composite reliability (CR) was evaluated, with values ≥ 0.7 considered acceptable^50^, though lower thresholds were allowed for exploratory constructs. Discriminant validity was assessed using the Fornell-Larcker criterion.

We estimated structural equation models using the lavaan package in R, applying Maximum Likelihood (ML**)** with Full Information Maximum Likelihood (FIML) to handle missing data. FIML allows the inclusion of all available data under the assumption that data are missing at random (MAR)^51^. This assumption was supported by Little’s MCAR test and visual inspection of the missingness patterns. To allow FIML to also address missingness in covariates, all control variables were treated as random variables, with their means, variances, and covariances freely estimated. FIML estimation was used for CFA and SEM models, while EFA was conducted on respondents with no missing values on relevant items.

The three latent factors, language proficiency, social integration, and economic and health-related concerns, were specified based on the results of prior exploratory and confirmatory factor analyses. We integrated them into Model 3. The latent variables were scaled by fixing their variances to 1, placing them on a standard deviation scale. In the CFA model, we allowed the covariance between the residuals of speaking ability and contacts with Germans, as stronger speaking skills may facilitate social interactions beyond what is captured by the latent structure. Similarly, in Model 3, we allowed the latent factors for language proficiency and social integration to covary. No equality constraints were imposed on the effects of integration barriers on life satisfaction. Standard errors and 95% confidence intervals were generated using 500 bootstrap resamples.

### Ethics approval and consent to participate

This study uses anonymized data from the 2022 IAB-BiB/FReDA-BAMF-SOEP Survey of Ukrainian Refugees in Germany. Survey procedures complied with the IAB Ethics Codex^52^ and followed ethical guidelines for research involving vulnerable populations affected by war and forced displacement. Participation in the original survey was voluntary and based on informed consent and data protection procedures ensuring confidentiality^53^. Respondents were informed that their participation would not influence their legal or residence status. To reduce risks of distress or re-traumatization, sensitive questionnaire items were carefully reviewed and pre-tested with Ukrainian refugees^42^.

## Results

### Descriptive results

We begin our analysis with the descriptive statistics of the study variables for separated and non-separated women (Table 1). Separated women (*M* = 5.71) report significantly lower life satisfaction than non-separated women (*M* = 5.96, Wilcoxon rank-sum test, *p* < 0.001). They also experience significantly higher loneliness (*M* = 2.00 vs. *M* = 1.30, *p* < 0.001). Separated women (*M* = 4.16) maintain more contact with non-relative Ukrainians (*M* = 3.86, *p* < 0.001) but not necessarily with Germans, suggesting a stronger reliance on the Ukrainian community for social interactions. Unexpectedly, perceived financial difficulties are slightly higher among those whose partners stayed in Germany (*M* = 3.81 vs. *M* = 3.69, *p* < 0.001). The proportion of women with higher education differs significantly between the two groups, with a higher percentage among separated women (73.5% vs. 68.2%, *p* < 0.001). However, employment status and language skills do not significantly differ. Women who left Ukraine and stayed in Germany without their partners are more likely to have children (87.0% vs. 78.5%, *p* < 0.001) and are less likely to intend to stay in Germany permanently or for the next several years (24.3% vs. 40.5%, *p* < 0.001). Spearman correlation coefficients are reported in Supplementary Table 1. Overall, the results suggest weak to moderate associations between variables (min ρ =  −0.28, max ρ = 0.79).

**Table 1:** Descriptive statistics.

	Range	Separated (partner in Ukraine), *N* = *3087*		Non-separated (partner in Germany), *N* = *2197*		*p*-value
		Mean or %	SD	Mean or %	SD	
Loneliness	0 – 4	2.0	1.25	1.3	1.26	< 0.001
Life satisfaction	0 – 10	5.71	1.94	5.96	1.97	< 0.001
Language proficiency in German*	1 – 5					
Speaking		1.66	0.79	1.65	0.79	0.433
Writing		1.73	0.88	1.74	0.88	0.455
Reading		2.09	1.01	2.07	1.02	0.390
Frequency of social contacts*	1 – 6					
With Germans		3.72	1.81	3.64	1.83	0.089
With non-relative Ukrainians		4.16	1.63	3.86	1.68	0.001
Feeling welcome in Germany*	1 – 5	4.02	0.95	4.00	0.95	0.501
Concerns* About your economic situation	1 – 3	2.23	0.56	2.24	0.57	0.932
About your health		1.83	0.59	1.85	0.58	0.502
Perceived financial difficulties*	1 – 6	3.69	1.05	3.81	1.07	0.001
Controls variables						
Age	18–71	40.13	10.22	41.28	13.33	0.898
Education:	0/1					0.001
High tertiary		73.5		68.2		
Lower		26.5		31.8		
Employment	0/1					0.344
Employed full-time/part-time		14.0		13.1		
Not employed/in education		86.0		86.9		
Having children	0/1					0.001
Yes		87.0		78.5		
No		13.0		21.5		
Intention to stay in Germany	0/1					0.001
Planning to stay for several years or permanently		24.3		40.5		
Planning to stay until the end of the war, for a maximum one year, or not sure		75.7		59.5		

The total sample size is 5,311 women. Information on separation is missing for 27 cases. Descriptive statistics are based on observed data. To ensure consistent interpretation, the original scales of these items were reverse-coded so that higher scores reflect higher levels of the respective construct.

### Factor structure of integration barriers

Through exploratory factor analysis (EFA), we identified a three-factor structure, namely language proficiency (F1), social integration (F2) and economic/health concerns (F3), which explained 46.4% of the total variance (Supplementary Table 2). The KMO index was 0.72, indicating acceptable sampling adequacy, and Bartlett’s test was significant (*χ*^*2*^(36) = 11,638.42, *p* < 0.001), supporting factorability of the data. We then tested these three factors in a confirmatory factor analysis (CFA) model, which confirmed their structure and showed an acceptable fit (CFI = 0.986; TLI = 0.978; RMSEA = 0.037; SRMR = 0.029) (Supplementary Table 3). Since factor loadings slightly differ when estimated within a full structural equation model, we report the standardized factor loadings from the final SEM model (Model 3), as they best reflect relationship within the theoretical framework (Table 2).

**Table 2:** Factor loadings for integration barriers.

Factor	Indicator	Standardized loading	*p*-value
Factor 1: Language proficiency			
	Speaking	0.74	< 0.001
	Writing	0.90	< 0.001
	Reading	0.84	< 0.001
Factor 2: Social integration			
	Contacts with Germans	0.62	< 0.001
	Contacts with non-relative Ukrainians	0.32	< 0.001
	Felling welcome	0.27	< 0.001
Factor 3: Economic and health concerns			
	Economic concerns	0.69	< 0.001
	Health concerns	0.31	< 0.001
	Perceived financial difficulties	0.51	< 0.001

For consistency, standardized factor loadings from Model 3 are reported here.* n* = 5,311. Fit statistics: *χ*^2^ (83) = 1124.47, *p* < 0.001, CFI = 0.932; TLI = 0.901; RMSEA = 0.049; SRMR = 0.035. Controlled for age, education, employment, accommodation, having kids, intention to stay in Germany. Prior to analysis, all original integration-barrier items were first reverse-coded so that higher scores reflect higher levels of the respective construct, and then z-standardized to ensure comparability across items measured on different scales.

The first factor, language proficiency, includes writing, reading, and speaking abilities in German, with strong standardized factor loadings (> 0.7). This factor demonstrates high reliability and validity, with composite reliability (CR) = 0.79, and average variance extracted (AVE) = 0.48, supporting its internal consistency and construct validity (Supplementary Table 4).

The second factor, social integration, encompasses contacts with Germans, contacts with non-relative Ukrainians and feeling welcome. Factor loadings vary, with contacts with Germans showing the strongest association (0.62), while contact with Ukrainians (0.32) and feeling being welcome (0.27). This factor exhibits low reliability and validity (CR = 0.55, AVE = 0.32), falling below conventional thresholds. The third factor, concerns, captures economic and health worries, and perceived financial difficulties, has moderate standardized factor loadings (ranging from 0.31 to 0.69). This factor shows marginally moderate reliability (CR = 0.68; AVE = 0.38), suggesting that while it does not fully meet strict reliability criteria, the construct remains meaningful within the model. All items loaded significantly into their respective factors. Discriminant validity is supported, as AVEs exceed squared inter-factor correlations (Supplementary Table 5).

Although factors 2 and 3 had low to moderate reliability, we retained them due to their theoretical importance and significant indirect effects in the SEM model. For example, interactions with non-relative Ukrainians may reflect the formation of new social support networks, which can be crucial when family ties are disrupted. Similarly, the feeling of being welcomed by the host community may serve as a key psychological buffer against the negative effects of separation. Given that our sample consists of refugees who arrived in Germany within the first 4 months of the war, early-stage integration processes may rely not only on interactions with the local Germans but also on connections within the Ukrainian refugee community.

### Model 1: Direct association between family separation and life satisfaction

To test our hypothesis H1, we run a first model, examining the direct association between family separation and life satisfaction. We found that family separation has a small but significant negative effect on life satisfaction (*b* =− 0.17, 95% CI [− 0.26, − 0.06], β =− 0.04, *p* = 0.001). This suggests that displaced Ukrainian women whose partners remain in Ukraine may report lower life satisfaction compared to those whose partner are in Germany. The explained variance in life satisfaction in Model 1 is relatively low (*R*^*2*^ = 5.0%).

### Model 2: Mediation through loneliness

**Fig. 1:**
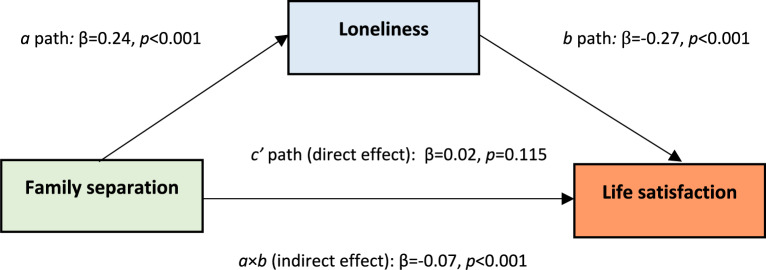
Model 2: Mediation model testing the association between family separation and life satisfaction, with loneliness as a single mediator. Standardized path coefficients are shown. We used 500 bootstrap samples in R to test whether loneliness mediates the relationship between forced family separation and life satisfaction (*n* = 5,311). Controlled for age, education, employment, accommodation, having kids, intention to stay in Germany.

In Model 2, we tested our hypothesis H2 by introducing loneliness as a single mediator of the relationship between family separation and life satisfaction (Fig. 1). The results show that a direct association between separation and life satisfaction (*c’* path) is no longer significant (*b* = 0.09, 95% CI [− 0.02, 0.20], β = 0.02, *p* = 0.115). Separation is strongly associated with increased loneliness (*a* path: *b* = 0.63, 95% CI [0.56, 0.70], β = 0.24, *p* < 0.001), and loneliness is in turn negatively associated with life satisfaction (*b* path: *b* =− 0.41, 95% CI [− 0.46, − 0.37], β =− 0.27, *p* < 0.001). The indirect effect (b =− 0.26, 95% CI [− 0.30, − 0.22], β =− 0.07, p < 0.001) is consistent with loneliness statistically fully mediating the association between separation and life satisfaction. This finding aligns with H2, suggesting that the connection between separation and life satisfaction is primarily channeled through loneliness, rather than a direct independent link. The inclusion of loneliness improved the explained variance in life satisfaction (*R*^*2*^ = 11.7%).

### Model 3: Serial-parallel mediation trough loneliness and integration barriers

**Fig. 2:**
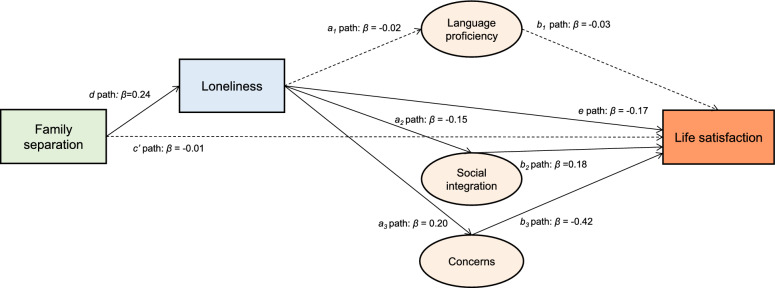
about here. Serial-parallel mediation SEM testing the association between family separation and life satisfaction trough loneliness and three integration-barrier factors.

In Model 3, we extended the analysis by incorporating three additional mediators that capture integration barriers: language proficiency (F1), social integration (F2), and concerns (F3). The results (Table 3 and Fig. 2.) indicate that the direct association between family separation and life satisfaction remains non-significant (*c’* path: *b* =− 0.03, 95% CI [− 0.12, 0.09], β =− 0.01, *p* = 0.642). This confirms the finding from Model 2 that separation does not show a direct link to life satisfaction. Instead, separation is associated with life satisfaction primarily through loneliness and subsequent integration challenges. Family separation is strongly associated with loneliness (*d* path: *b* = 0.63, 95% CI [0.56, 0.70], β = 0.24, *p* < 0.001), and loneliness remains a significant negative correlate of life satisfaction (*e* path: *b* =− 0.24, 95% CI [− 0.30, − 0.19], β =− 0.17, *p* < 0.001), demonstrating its essential intermediary role.

**Table 3:** Results of the serial-parallel mediation SEM model examining the association between family separation and life satisfaction through loneliness and integration barriers: Model 3.

Pathway	Estimate (b)	SE	*p*-Value	Bootstrap 95% CI	Standardized (β)
Direct effects					
Separation → Life satisfaction: *c’* path	− 0.03	0.06	0.642	[− 0.12; 0.09]	− 0.01
Loneliness → Life satisfaction: *e* path	− 0.24	0.03	< 0.001	[− 0.30; − 0.19]	− 0.17
Separation → Loneliness: *d* path	0.63	0.04	< 0.001	[0.56; 0.70]	0.24
Loneliness → F1 (Language proficiency): *a*_*1*_ path	− 0.01	0.01	0.231	[− 0.04; 0.01]	− 0.02
Loneliness → F2 (Social integration): *a*_*2*_ path	− 0.12	0.02	< 0.001	[− 0.16; − 0.08]	− 0.15
Loneliness → F3 (Economic and health concerns): *a*_*3*_ path	0.16	0.02	< 0.001	[0.13; 0.19]	0.20
F1 (Language proficiency): → Life satisfaction: *b*_*1*_ path	− 0.05	0.03	0.055	[− 0.12; 0.01]	− 0.03
F2 (Social integration): → Life satisfaction: *b*_*2*_ path	0.33	0.05	< 0.001	[0.22; 0.44]	0.17
F3 (Economic and health concerns) → Life satisfaction: *b*_*3*_ path	− 0.76	0.04	< 0.001	[− 0.83; − 0.69]	− 0.42
Indirect effects					
Separation → Loneliness → Life satisfaction	− 0.15	0.02	< 0.001	[− 0.19; − 0.12]	− 0.04
Separation → Loneliness → F1 (Language proficiency) → Life satisfaction	0.001	0.001	0.399	[− 0.0; 0.002]	0.00
Separation → Loneliness → F2 (Social integration) → Life satisfaction	− 0.03	0.01	0.001	[− 0.05; − 0.02]	− 0.01
Separation → Loneliness → F3 (Economic and health concerns) → Life satisfaction	− 0.07	0.01	< 0.001	[− 0.10; − 0.06]	− 0.02
Total indirect effect	− 0.25	0.02	< 0.001	[− 0.29; − 0.25]	− 0.06
Total effect: direct (*c’* path) + total indirect	− 0.28	0.05	0.001	[− 0.38; − 0.17]	− 0.07

*n* = 5,311. R^2^ = 29.0%. Fit statistics: *χ*^2^ (83) = 1124.47, *p* < 0.001, CFI = 0.932, TLI = 0.901, RMSEA = 0.049, SRMR = 0.035. Controlled for age, education, employment, accommodation, having kids, intention to stay in Germany. F1, F, F3 – integration barrier factors: F1—Language proficiency; F2—Social integration; F3—Economic and health concerns. Prior to analysis, all original integration-barrier items were first reverse-coded (so that higher scores reflect higher levels of the respective construct), and z-standardized to ensure comparability across items measured on different scales. Bootstrapping with 500 resamples was used to generate standard errors and 95% confidence intervals; all *p*-values are two-tailed and based on z statistics derived from bootstrapped standard errors.

Next, among the three latent mediators, factor 3 (economic and health concerns), is the strongest factor associated with reduced life satisfaction (*b*_*3*_ path:* b* =− 0.76, 95% CI [− 0.83, − 0.69], β =− 0.42, *p* < 0.001), followed by social integration (factor 2) (*b*_*2*_ path: *b* = 0.33, 95% CI [0.22, 0.44], β = 0.17, *p* < 0.001) (Fig. 2). Social integration is positively related to life satisfaction, whereas economic and health concerns are linked to its decline. Language proficiency (factor 3), however, does not show a significant association with life satisfaction (*b*_*1*_ path: *b* =− 0.05, 95% CI [− 0.12, 0.01], β =− 0.03, *p* = 0.055). Furthermore, loneliness is negatively associated with social integration (*a*_*2*_ path: *b* =− 0.12, 95% CI [− 0.16, − 0.08], β =− 0.15, *p* < 0.001) and positively linked with economic and health concerns (*a*_*3*_ path: *b* =− 0.16, 95% CI [0.13, 0.19], β = 0.20, *p* < 0.001). This pattern suggests its important role in observed associations where social connectedness appears weaker and economic and health vulnerabilities are higher when loneliness is present.

Our findings show that loneliness is not only directly associated with lower life satisfaction but also indirectly linked to it through increased economic and health concerns and reduced social integration. Specifically, the total indirect effect of separation on life satisfaction through loneliness and integration barriers is statistically significant (*b* =− 0.25, 95% CI [− 0.29, − 0.21], β =− 0.06, *p* < 0.001) and accounts for a large proportion of the total effect (*b* =− 0.28, 95% CI [− 0.38, − 0.17], β =− 0.07, *p* < 0.001). This supports Hypothesis 3, highlighting the intermediary role of loneliness in the association between family separation and life satisfaction. As shown in Table 4, nearly the entire association between separation on life satisfaction appears to be accounted for by loneliness and related pathways. The largest portion of the total indirect effect is linked to the direct path from loneliness to life satisfaction (59.7%). The path through loneliness and economic and health concerns accounts for 32.3%, while the path through loneliness and social integration contributes 8.1%. Language proficiency does not significantly mediate this relationship. Compared to Model 2, the explained variance in life satisfaction increased notably (R^2^ = 29%).

**Table 4:** Contribution of indirect pathways to the total indirect effect of family separation on life satisfaction.

Pathway	Standardized (β)	% contribution to total standardized indirect effect
Separation → Loneliness → Life satisfaction	− 0.04	59.7
Separation → Loneliness → F1 (Language proficiency) → Life satisfaction	0.00	0.0
Separation → Loneliness → F2 (Social integration) → Life satisfaction	− 0.01	8.1
Separation → Loneliness → F3 (Economic and health concerns) → Life satisfaction	− 0.02	32.3
Total indirect effect	− 0.06	100.0

Percentage contributions represent the proportion of the total indirect effect explained by each pathway. *n* = 5,311. Fit statistics: *χ*^2^ (83) = 1124.47, *p* < 0.001, CFI = 0.932, TLI = 0.901, RMSEA = 0.049, SRMR = 0.035. Controlled for age, education, employment, accommodation, having kids, intention to stay in Germany. F1, F, F3 – integration barrier factors, F1—Language proficiency; F2—Social integration; F3—Economic and health concerns. Prior to analysis, all original integration-barrier items were first reverse-coded so that higher scores reflect higher levels of the respective construct, and then z-standardized to ensure comparability across items measured on different scales.

### Robustness checks

To assess the robustness of our findings, we estimated two alternative models: Models 4 and 5 (Supplementary Tables 6 and 7). Models 4 examines whether loneliness in our study is primarily related to forced partner separation rather than reflecting broader displacement challenges. It has the same specification as Model 3 but compares separated partnered women to single women (Supplementary Table 6). If our measure captures separation-related distress, the association between partner absence and loneliness should be stronger in Model 3 (separated vs. non-separated partnered women) than in Model 4 (separated partnered vs. single women). Consistent with this, the association between partner absence and loneliness was substantially weaker in Model 4 (b = 0.14, 95% CI [0.05, 0.24], β = 0.05, p = 0.004) than in Model 3 (b = 0.63, 95% CI [0.56, 0.70], β = 0.24, *p* < 0.001). The indirect effect on life satisfaction via loneliness was also smaller (b =− 0.03, 95% CI [− 0.06, − 0.01], β =− 0.008, *p*  = 0.006 vs. b =− 0.15, 95% CI [− 0.19, − 0.12], β =− 0.04, *p* =  < 0.001). These results suggest that loneliness in our study is more closely related to emotional distress linked to forced separation then to general resettlement issues.

Model 5 test an alternative directionality, where integration barriers precede loneliness, keeping all other paths as in Model 3 (Supplementary Table 7). In this model, social integration (β = –0.16, *p* < 0.001) and economic and health concerns (β = 0.20, p < 0.001) are significantly linked to loneliness, with magnitude similar to Model 3. The main indirect effect still operates through loneliness (b = –0.27, 95% CI [–0.32, –0.23], β = –0.07, *p* < 0.001). However, among integration-related paths, only the one via social integration (Factor 2) remains significant, and it is very small (b = 0.01, 95% CI [0.004, 0.027], β = 0.003, *p*  = 0.026). Compared with this alternative, Model 3 provides a better fit (CFI = 0.932 vs. 0.881; RMSEA = 0.049 vs. 0.060) and explains more variance in life satisfaction (R^2^ = 29.0% vs. 11.5%). While we cannot establish causal direction with our cross-sectional data, there results indicate that our main model better represents the observed associations between forced separation, loneliness, integration barriers, and life satisfaction.

In addition, we estimated a series of ordinary least squares (OLS) regression models to examine the mediation pathways proposed in Model 3 using a standard frequentist approach (Supplementary Tables 8 and 9). The OLS results are highly consistent with the SEM findings, confirming the central role of loneliness and the significance of indirect effects via social integration and economic and health concerns, while the direct effect of family separation on life satisfaction remains non-significant.

## Discussion

This study examined how war-related forced family separation, particularly the involuntary separation of Ukrainian refugee women from their partners due to wartime mobility restrictions, is associated with loneliness, integration challenges, and life satisfaction during early resettlement in Germany. The experience of Ukrainian displaced women differs from that of other refugee groups, because most refugee women who arrived between 2013 and 2016 were more likely to flee with their families, including partners^54^. For these earlier groups Gambaro et al.^54^ reported life satisfaction scores of 6.1 for refugees with spouses remained abroad and 7.5 for those whose spouses were in Germany. In comparison, our study found lower life satisfaction scores among Ukrainian women: 5.71 for those whose partner remained in Ukraine and 5.96 for those whose partner stayed in Germany. The findings suggest that Ukrainian women who arrived in 2022 experienced quite low life satisfaction despite certain advantages, such as facilitated legal status and easier access to services^55^.

Our study contributes to the literature in three main ways. First, we conceptualize loneliness as an immediate emotional response to forced partner separation, reflecting the distress women experience being apart from their partners who remain in a war-torn country. This interpretation aligns with Weiss’ (1973) theoretical distinction between emotional and social loneliness when emotional loneliness arises from the absence of a close attachment figure^19^. Although the single-item loneliness measure we used may reflect broader post-displacement stressors, three aspects support out interpretation: 1) the involuntary nature of partner separation; 2) the timing of data collection shortly after participants’ displacement to Germany, and 3) the robustness check in Model 4, which shows a much stronger association between partner absence and loneliness when comparing separated and non-separated partnered women than comparing separated partnered and single women. In addition, prior research shows that single-item measures of loneliness can serve as valid and trustworthy tool in loneliness research^56^.

Second, we examine how this form of loneliness may be linked to early integration challenges among partnered refugee women, an aspect that has received limited attention in prior research. Whereas many studies report that integration difficulties are linked to greater loneliness^10,18,38^, we highlight the reverse pathway: emotional loneliness may be associated with greater perceived barriers to integration. Drawing on stress theory and emotional-cognitive processing models^57^, we suggest that in uncontrollable situations such as war-related family separation, emotion-focused coping may be more prevalent and associated with lower psychological and cognitive resources for addressing integration challenges. Such directionality reflects a possible sequence from immediate emotional distress to gradually developing social and practical difficulties, as lonely individuals often have lower enthusiasm for social interactions (e.g. in adolescents^58^), heightened fears for rejection^59^ and reduced motivation to learn a new language and build social networks^39,60,61^. The results of the alternative Model 5 indicate significant associations when integration factors are specified as preceding loneliness. However, our preferred Model 3 provides a better overall fit, explains more variance in life satisfaction, and highlights a stronger role of loneliness as an intermediary factor.

Third, we contribute to research on refugee well-being^9,21,62^ by showing that the link between forced family separation and life satisfaction is mediated by loneliness and integration barriers rather than being direct. Our findings also suggest that a higher level of social integration is positively associated with life satisfaction. This is consistent with previous research highlighting the importance of social networks in promoting refugee well-being, particularly contacts with locals^9,34,63^. We also found that economic and health concerns are linked to lower life satisfaction, consistent with prior studies^34,37^. Surprisingly, we do not find a statistically significant association between the language factor and life satisfaction. This finding contradicts the previous research suggesting that better language skills improve refugee well-being^2,34^. A possible explanation is the limited variation in German language proficiency between separated and non-separated Ukrainian refugee women at the early stages of resettlement, as well as the mitigating effect of strong Ukrainian immigrant networks providing social support and connections^64^. Overall, our results align with previous studies highlighting loneliness as strongly associated with lower well-being^11,65^ and as a major concern among Ukrainian refugees in other countries, such as the Czech Republic^30^ and Poland^31^.

### Implications

Our findings suggest that integration policies and programs should place greater focus on the mental well-being of war refugees, particularly vulnerable groups such as forcibly separated women. Monitoring various emotional states, including loneliness, through survey-based approaches may help identify early risks for subsequent integration difficulties and other social challenges among refugee populations. Overall, this nuanced evidence may also improve the evaluation of existing integration policies and programs. More tailored intersectoral policies are needed to design and implement interventions aimed at reducing loneliness, which may, in turn, facilitate better social integration during the early stages of resettlement. These policies should focus on improving access to language education, expanding job opportunities, fostering social networks and increasing access to mental health services.

### Limitations

Our study has several limitations. First, our main outcome variable, life satisfaction, was assessed using a single-item global life satisfaction Likert scale. This approach was chosen for three main reasons. First, the single-item measure is widely used in large-scale national and international surveys, including the European Social Survey, World Values Survey, the German Socio-Economic Panel (SOEP), to capture global life satisfaction. Second, as this is a secondary data analysis, we were limited to the variables collected in the original survey design, which did not include a multi-item scale for life satisfaction. Third, previous research has shown that it demonstrates acceptable levels of reliability and validity^66^.

Next, we acknowledge that the three integration-barrier factors do not capture the full complexity of the integration process, and two of them, Factors 2 and 3, have suboptimal reliability, indicating reduced explanatory power^50^. Despite this, we retained the factors for both theoretical and empirical reasons. The three factors were developed specifically for this study and therefore serve an exploratory role in capturing early-stage integration difficulties.

Factor 1 (language proficiency), although often considered part of social integration, was treated as a separate dimension due its central role in early communication. In Model 3, we specified a covariance between Factors 1 and 2 to account for the link between language skills and social integration opportunities. However, language proficiency relies on self-reported measures, which may lead to over- or underestimation of actual skills. Factor 2 (social integration) reflects early-stage resettlement social networks and feelings of being welcome, both of which can facilitate adaptation shortly after displacement. Nevertheless, it does not capture the quality of these interactions, and is based on the limited number of items. Factor 3 (economic and health worries) captures some elements of economic integration and psychological well-being but primarily reflects perceived constraints that may hinder integration. While acknowledging their limitations, we believe that the three factors reflect important aspects of early-stage integration among displaced women, and including them in the Model 3 revealed meaningful indirect pathways through which loneliness is associated with life satisfaction. Moreover, SEM can account for some degree of unreliability in observed indicators**,** making the estimates more robust to moderate measurement error^67^.

Another limitation is the cross-sectional design of the study. Model 3 assumes certain directional relationships, including loneliness preceding integration factors, that cannot be definitively verified without longitudinal data. Moreover, separation is not necessarily static. Some women may have reunited with their partners in Germany or temporarily reconnected with them during visits to Ukraine over the course of the war^68^. We cannot not capture these patterns with the cross-sectional data. However, incorporating longitudinal data would exceed the scope of the paper, and the split of the IAB-BiB/FReDA-BAMF-SOEP Survey after the second wave would make panel analyses possible only at the cost of substantially reduced sample size. Therefore, longitudinal studies are essential to capture changes in separation status and their effects on Ukrainian refugees’ subjective well-being over time.

Finally, this study primarily focuses on the interplay between separation, loneliness and integration difficulties, but other factors, such as education, children, and experiences of war, may also play a role. Our Model 3 results for the control variables show that higher education is positively associated with two integration-related factors (Factor 1: β = 0.098, p < 0.001, and Factor 2: β = 0.062, p = 0.003), but also linked to lower life satisfaction (β =− 0.034, p = 0.01). This is consistent with the “status dissonance” explanation, when highly qualified refugees experience frustration due to mismatches between their skills and available jobs^69^. In contrast, having children is associated with lower loneliness (β =− 0.03, p = 0.032), and higher life satisfaction (β = 0.038, p = 0.013). It also shows negative associations with Factor 1 (β =− 0.08, p < 0.001), and Factor 3 (β =− 0.06, p = 0.005), possibly reflecting caregiving-related constraints. After displacement and separation, many Ukrainian women become sole caregivers, facing challenges in balancing family duties with language learning and other integration demands. In addition, we lack information on the quality of relationship between partners and on personality traits, both of which may impact partnered women’s vulnerability to emotional loneliness. Future research should explore how different factors and dynamics, including progress in language acquisition, intentions to stay or return, plans for family reunification evolve over time as displaced women further integrate into host community, and how these changes relate to their life satisfaction.

## Conclusions

This study shows that war-related forced family separation is significantly associated with lower life satisfaction among Ukrainian refugee women, with this relationship mediated by loneliness and integration barriers. By identifying loneliness as a distinct and important pathway linking involuntary separation to lower well-being, our findings contribute to a broader understanding of how emotional and social factors may interact and relate to subjective well-being of displaced women. While causal claims cannot be made due to the cross-sectional design, our findings suggest that loneliness not only accounts for the largest portion of the association between separation and life satisfaction, but also appears to play an intermediary role by linking forced separation to greater integration challenges and lower life satisfaction. This perspective is especially relevant for newly arrived refugees. Overall, these findings highlight the importance of considering emotional well-being in relation to structural integration processes when examining refugee life satisfaction.

## Supplementary Information

Supplementary Information 1.

Supplementary Information 2.

## Data Availability

The data analyzed in this study are publicly available from the Research Data Center SOEP at the German Institute for Economic Research (DIW Berlin). The dataset used is the IAB-BiB/FReDA-BAMF-SOEP Survey “Refugees from Ukraine in Germany”, 1st wave, 2022, and can be accessed upon request via the SOEP Research Data Center: https://www.diw.de/en/diw_01.c.357906.en/soep_order_form.html.

## Code availability

The R code used to generate the estimates reported in this study is available from the corresponding author upon request.
