# Supplementary material for: Loneliness as a mediator between family separation and life satisfaction among Ukrainian refugee women in Germany
Source: Sci Rep. 2026 May 30;16:16803. doi: 10.1038/s41598-026-55664-w (PMC13226654; doi:10.1038/s41598-026-55664-w)
Supplement: Supplementary file 1 — Supplementary Information 1. [file 41598_2026_55664_MOESM1_ESM.pdf]

# Loneliness as a mediator between family separation and life satisfaction among Ukrainian refugee women in Germany

*Nataliia Levchuk[1,2,3], Domantas Jasilionis [1,4], Lisa Kriechel[2], Martin Bujard[2,5]*

[1] Max Planck Institute for Demographic Research (MPIDR), Rostock, Germany
[2] Federal Institute for Population Research (BIB), Wiesbaden, Germany
[3] Mykhailo Ptukha Institute for Demography and Life Quality Research, Kyiv, Ukraine
[4] Max Planck - University of Helsinki Center for Social Inequalities in Population Health (MaxHel Center), Rostock, Germany
[5] Institute of Medical Psychology, Medical Faculty, University Heidelberg, Germany

# Supplementary Information

## Table of contents

**Supplementary Table 1.** Spearman correlations between study variables (provided as a separate file titled “Supplementary Table 1”).

**Supplementary Table 2.** Factor loadings from exploratory factor analysis (EFA)

**Supplementary Table 3.** Standardized factor loadings from confirmatory factor analysis (CFA)

**Supplementary Table 4.** Composite reliability (CR)

**Supplementary Table 5.** Discriminant validity (Fornell-Larcker Criterion)

**Supplementary Table 6.** Results of the serial-parallel mediation SEM model testing the association between partner absence and life satisfaction through loneliness and integration barriers, comparing separated partnered women with those who are single or not in stable relationships (Model 4).

**Supplementary Table 7.** Results of the serial-parallel mediation SEM model testing the association between family separation and life satisfaction, where the three integration-barrier factors precede loneliness (Model 5).

**Supplementary Table 8.** OLS mediation results.

**Supplementary Table 9.** OLS mediation effects (bootstrapped).

**Supplementary Table 2. Factor loadings from exploratory factor analysis (EFA)**

| Variable | Factor 1 Language proficiency in German | Factor 2 Social integration | Factor3 Concerns | Communality ($h^2$) |
|---|---|---|---|---|
| Language proficiency | | | | |
| - Speaking | 0.77 | | | 0.608 |
| - Writing | 0.94 | | | 0.866 |
| - Reading | 0.87 | | | 0.759 |
| Social integration | | | | |
| - Contacts with Germans | | 0.99 | | 0.990 |
| - Contacts with non-relative Ukrainians | | 0.26 | | 0.149 |
| - Felling welcome | | 0.18 | | 0.149 |
| Concerns | | | | |
| - Economic concerns | | | 0.71 | 0.488 |
| - Health concerns | | | 0.29 | 0.191 |
| - Perceived financial difficulties | | | 0.53 | 0.281 |

*Note:* Prior to analysis, all original integration-barrier items were first reverse-coded so that higher scores reflect higher levels of the respective construct, and then z-standardized to ensure comparability across items measured on different scales.

**Supplementary Table 3. Standardized factor loadings from confirmatory factor analysis (CFA)**

| Factor | Indicator | Standardized loading (Std.all) | p-value |
|---|---|---|---|
| Factor 1: Language proficiency | | | |
| | Speaking | 0.78 | <0.001 |
| | Writing | 0.93 | <0.001 |
| | Reading | 0.87 | <0.001 |
| Factor 2: Social integration | | | |
| | Contacts with Germans | 0.82 | <0.001 |
| | Contacts with non-relative Ukrainians | 0.27 | <0.001 |
| | Felling welcome | 0.24 | <0.001 |
| Factor 3: Economic and health concerns | | | |
| | Economic concerns | 0.74 | <0.001 |
| | Health concerns | 0.30 | <0.001 |
| | Perceived financial difficulties | 0.50 | <0.001 |

*Note:* Prior to analysis, all original integration-barrier items were first reverse-coded so that higher scores reflect higher levels of the respective construct, and then z-standardized to ensure comparability across items measured on different scales. Fit statistics: CFI=0.986; TLI=0.978; RMSEA=0.037; SRMR=0.030.

**Supplementary Table 4. Composite reliability (CR)**

| Factor | Composite reliability | Average variance extracted |
|---|---|---|
| Factor 1: Language proficiency | 0.79 | 0.48 |
| Factor 2: Social integration | 0.55 | 0.32 |
| Factor 3: Economic and Concerns | 0.68 | 0.38 |

**Supplementary Table 5. Discriminant validity (Fornell-Larcker Criterion)**

| Factor | Factor 1 | Factor 2 | Factor 3 |
|---|---|---|---|
| Factor 1: Language proficiency | 0.69 | 0.28 | -0.11 |
| Factor 2: Social integration | 0.28 | 0.56 | -0.05 |
| Factor 3: Economic and health concerns | -0.11 | -0.05 | 0.62 |

**Supplementary Table 6. Results of the serial-parallel mediation SEM model testing the association between partner absence and life satisfaction through loneliness and integration barriers, comparing separated partnered women with those who are single or not in stable relationships (Model 4).**

| Pathway | Estimate (b) | SE | p-value | Bootstrap 95% CI | Standardized (β) |
|---|---|---|---|---|---|
| **Direct effects:** | | | | | |
| Partner absence → Life satisfaction: *c′* path | -0.07 | 0.07 | 0.322 | [-0.21; 0.07] | -0.02 |
| Loneliness → Life satisfaction: *e* path | -0.23 | 0.03 | <0.001 | [-0.29; -0.16] | -0.15 |
| Partner absence → Loneliness: *d* path | 0.14 | 0.05 | 0.004 | [0.05; 0.24] | 0.05 |
| Loneliness → F1 (Language proficiency): $a_1$ path | -0.02 | 0.01 | 0.07 | [-0.05; 0.00] | -0.03 |
| Loneliness → F2 (Social integration): $a_2$ path | -0.17 | 0.03 | <0.001 | [-0.24; -0.12 | -0.20 |
| Loneliness → F3 (Economic and health concerns): $a_3$ path | 0.20 | 0.02 | <0.001 | [0.17; 0.24] | 0.24 |
| F1 (Language proficiency): → Life satisfaction: $b_1$ path | 0.02 | 0.04 | 0.59 | [-0.06; 0.09] | 0.01 |
| F2 (Social integration): → Life satisfaction: $b_2$ path | 0.29 | 0.07 | <0.001 | [0.18; 0.44] | 0.16 |
| F3 (Economic and health concerns) → Life satisfaction: $b_3$ path | -0.78 | 0.04 | <0.001 | [-0.85; -0.71] | -0.42 |
| **Indirect effects:** | | | | | |
| Partner absence → Loneliness → Life satisfaction | -0.03 | 0.00 | 0.006 | [-0.06; -0.01] | -0.008 |
| Partner absence → Loneliness → F1 (Language proficiency) → Life satisfaction | -0.000 | 0.004 | 0.673 | [-0.0; 0.00] | -0.00 |
| Partner absence →Loneliness → F2 (Social integration) →Life satisfaction | -0.007 | 0.008 | 0.066 | [-0.02; -0.01] | -0.002 |
| Partner absence →Loneliness →F3 (Economic and health concerns) →Life satisfaction | -0.022 | 0.011 | 0.005 | [-0.04; -0.01] | -0.01 |
| Total indirect effect | -0.06 | 0.021 | 0.004 | [-0.1; -0.02] | -0.02 |
| **Total effect: direct (*c′* path) + total indirect** | -0.128 | 0.072 | 0.075 | [-0.27; 0.01] | -0.03 |

*n*=5,121. $R^2$=29.7%. Fit statistics: $\chi^2$ (83)=1014.20, p<0.001, CFI=0.937, TLI=0.901, RMSEA=0.047, SRMR=0.035. Controlled for age, education, employment, accommodation, having kids, intention to stay in Germany.

*Note:* F1, F, F3 – integration barrier factors: F1 - Language proficiency; F2 - Social integration; F3 - Economic and health concerns. Prior to analysis, all original integration-barrier items were first reverse-coded (so that higher scores reflect higher levels of the respective construct), and z-standardized to ensure comparability across items measured on different scales. Bootstrapping with 500 resamples was used to generate standard errors and 95% confidence intervals; all p-values are two-tailed and based on z statistics derived from bootstrapped standard errors.

**Supplementary Table 7. Results of the serial-parallel mediation SEM model testing the association between family separation and life satisfaction, where the three integration-barrier factors precede loneliness (Model 5).**

| **Pathway** | Estimate (b) | SE | p-value | Bootstrap 95% CI | Standardized (β) |
|---|---|---|---|---|---|
| **Direct effects:** | | | | | |
| Separation → Life satisfaction: *c'* path (direct effect) | 0.09 | 0.06 | 0.100 | [-0.02; 0.19] | 0.02 |
| Loneliness → Life satisfaction | -0.41 | 0.02 | <0.001 | [-0.46; -0.36] | -0.27 |
| Separation → Loneliness | 0.67 | 0.04 | <0.001 | [0.59; 0.75] | 0.26 |
| Separation → F1 (Language proficiency) | 0.05 | 0.03 | 0.102 | [-0.01; 0.11] | 0.02 |
| Separation → F2 (Social integration) | 0.16 | 0.05 | 0.001 | [0.27; 0.16] | 0.08 |
| Separation → F3 (Economic and health concerns) | -0.06 | 0.04 | 0.090 | [-0.14; 0.01] | -0.03 |
| F1 (Language proficiency): → Loneliness | 0.04 | 0.02 | 0.058 | [0.0; 0.09] | 0.03 |
| F2 (Social integration): → Loneliness | -0.2 | 0.04 | <0.001 | [-0.28; -0.13] | -0.16 |
| F3 (Economic and health concerns) → Loneliness | 0.26 | 0.03 | <0.001 | [0.21; 0.31] | 0.20 |
| **Indirect effects:** | | | | | |
| Separation → Loneliness → Life satisfaction | -0.27 | 0.02 | <0.001 | [-0.32; -0.23] | -0.07 |
| Separation → F1 (Language proficiency) → Loneliness → Life satisfaction | -0.001 | 0.001 | 0.272 | [-0.003; 0.0] | 0.00 |
| Separation → F2 (Social integration) → Loneliness →Life satisfaction | 0.01 | 0.01 | 0.026 | [0.004; 0.027] | 0.003 |
| Separation →F3 (Economic and health concerns) →Loneliness→ Life satisfaction | 0.01 | 0.01 | 0.104 | [-0.001; 0.015] | 0.002 |
| Total indirect effect | -0.26 | 0.02 | <0.001 | [-0.299; -0.214] | -0.06 |
| **Total effect: direct (*c'* path) + total indirect** | -0.17 | 0.05 | 0.002 | [-0.273; -0.062] | -0.04 |

*n*=5,311. $R^2$=11.5%. Fit statistics: $\chi^2$ (83)=1675.188, *p*<0.001, CFI=0.881, TLI=0.820, RMSEA=0.060, SRMR=0.044. Controlled for age, education, employment, accommodation, having kids, intention to stay in Germany.

*Note:* F1, F, F3 – integration barrier factors: F1 - Language proficiency; F2 - Social integration; F3 - Economic and health concerns. Prior to analysis, all original integration-barrier items were first reverse-coded (so that higher scores reflect higher levels of the respective construct), and z-standardized to ensure comparability across items measured on different scales. Bootstrapping with 500 resamples was used to generate standard errors and 95% confidence intervals; all p-values are two-tailed and based on z statistics derived from bootstrapped standard errors.

## Supplementary Table 8. OLS mediation results

| Pathway | Estimate (b) | SE | p-value | 95% CI |
|---|---|---|---|---|
| Separation → Life satisfaction: *c′* path (direct effect) | -0.004 | 0.05 | 0.947 | [-0.11; 0.09] |
| Loneliness → Life satisfaction: *e* path | -0.32 | 0.02 | <0.001 | [-0.36; -0.29] |
| Separation → Loneliness: *d* path | 0.63 | 0.04 | <0.001 | [0.56; 0.70] |
| Loneliness → F1 (Language proficiency): $a_1$ path | -0.02 | 0.01 | 0.07 | [-0.04; 0.002] |
| Loneliness → F2 (Social integration): $a_2$ path | -0.07 | 0.01 | <0.001 | [-0.08; -0.05] |
| Loneliness → F3 (Economic and health concerns): $a_3$ path | 0.10 | 0.01 | <0.001 | [0.08; 0.12] |
| F1 (Language proficiency): → Life satisfaction: $b_1$ path | -0.04 | 0.03 | 0.18 | [-0.09; 0.02] |
| F2 (Social integration): → Life satisfaction: $b_2$ path | 0.24 | 0.03 | <0.001 | [0.18; 0.31] |
| F3 (Economic and health concerns) → Life satisfaction: $b_3$ path | -0.72 | 0.03 | <0.001 | [-0.78; -0.66] |

*n* = 5,144; $R^2 = 0.208$; adjusted $R^2 = 0.206$; $F(11, 5132) = 122.2$, $p < 0.001$. Controlled for age, education, employment, accommodation, presence of children, intention to stay in Germany.

*Note:* Coefficients are estimated using ordinary least squares (OLS) regression. OLS models are based on listwise deletion of missing values, whereas the SEM models were estimated using full information maximum likelihood (FIML).

## Table 9. OLS mediation effects (bootstrapped)

| Effects: | Estimate | 95% CI |
|---|---|---|
| Separation → Loneliness → Life satisfaction | -0.21 | [-0.24; -0.17] |
| Separation → Loneliness → F1 (Language proficiency) → Life satisfaction | 0.00 | [-0.0; 0.001] |
| Separation →Loneliness → F2 (Social integration) →Life satisfaction | -0.01 | [-0.01; -0.01] |
| Separation →Loneliness →F3 (Economic and health concerns) →Life satisfaction | -0.05 | [-0.06; -0.04] |
| Total indirect effect | -0.260 | [-0.30; -0.22] |
| Direct effect | -0.004 | [-0.11; 0.09] |
| Total effect: direct + total indirect | -0.263 | [-0.37; -0.16] |

*Note:* Indirect effects are computed using OLS-based mediation analysis. Bootstrapped standard errors and 95% confidence intervals are based on 2,000 resamples. All models include the same control variables as in Table 8.
