# Supplementary material for: Loneliness as a mediator between family separation and life satisfaction among Ukrainian refugee women in Germany
Source: Sci Rep. 2026 May 30;16:16803. doi: 10.1038/s41598-026-55664-w (PMC13226654; doi:10.1038/s41598-026-55664-w)
Supplement: Supplementary file 2 — Supplementary Information 2. [file 41598_2026_55664_MOESM2_ESM.pdf]

Supplementary Table 1. Spearman correlations (two-sided) between study variables

	1	2	3	4	5	6	7	8	9	10	11	12	13	14	15	16
1. Life satisfaction	-															
	-															
2. Loneliness	-0.28															
	p<0.001															
3. Language proficiency in German: Speaking	0.08	-0.01														
	p<0.001	p=0.28														
4. Language proficiency in German: Writing	0.06	-0.03	0.72													
	p<0.001	p<0.05	p<0.001													
5.Language proficiency in German: Reading	0.06	-0.02	0.68	0.79												
	p<0.001	p=0.11	p<0.001	p<0.001												
6. Frequency of social contacts with Germans	0.12	-0.08	0.25	0.21	0.21											
	p<0.001	p<0.001	p<0.001	p<0.001	p<0.001											
7. Frequency of social contacts with non-relative Ukrainians	0.01	-0.05	0.07	0.1	0.11	0.21										
	p=0.31	p<0.001	p<0.001	p<0.001	p<0.001	p<0.001										
8. Feeling welcome in Germany	0.22	-0.13	0.05	0.04	0.03	0.15	0.02									
	p<0.001	p<0.001	p<0.001	p=0.002	p=0.022	p<0.001	p=0.165									
9. Concerns about your economic situation	-0.3	0.12	-0.05	-0.05	-0.05	-0.02	0.01	-0.09								
	p<0.001	p<0.001	p<0.001	p=0.001	p<0.001	p=0.129	p=0.331	p<0.001								
10. Concerns about your health	-0.22	0.11	-0.09	-0.09	-0.09	-0.03	-0.03	-0.07	0.21							
	p<0.001	p<0.001	p<0.001	p<0.001	p<0.001	p=0,017	p=0,015	p<0.001	p<0.001							
11. Perceived financial difficulties	-0.23	0.07	-0.04	-0.06	-0.08	-0.01	0.01	-0.14	0.36	0.13						
	p<0.001	p<0.001	p<0.001	p<0.001	p<0.001	p=0.308	p=0.336	p<0.001	p<0.001	p<0.001						
12. Separation	-0.06	0.27	0.01	-0.01	0.01	0.02	0.09	0.01	0.01	-0.01	-0.05					
	p<0.001	p<0.001	p=0.432	p=0.455	p=0.391	p=0.089	p<0.001	p=0.501	p=0.932	p=0.502	p<0.001					

	1	2	3	4	5	6	7	8	9	10	11	12	13	14	15	16
13. Age	-0.07	-0.14	-0.15	-0.13	-0.16	-0.06	0.02	0.08	0.07	0.22	0.04	-0.05				
	p<0.001	p<0.001	p<0.001	p<0.001	p<0.001	p<0.001	p=0,09	p<0.001	p<0.001	p<0.001	p=0.003	p<0.001				
14. Education	-0.04	0.04	0.09	0.08	0.09	0.04	0.02	-0.02	0.04	-0.05	0.01	0.06	-0.05			
	p=0.007	p=0.002	p<0.001	p<0.001	p<0.001	p=0.002	p=0,216	p=0,269	p=0,007	p<0.001	p=0.616	p<0.001	p<0.001			
15. Employment	0.03	0.02	0.12	0.1	0.1	0.15	-0.03	0.02	-0.09	-0.05	-0.04	0.01	-0.1	0.05		
	p=0.030	p=0.229	p<0.001	p<0.001	p<0.001	p<0.001	p=0.015	p=0.111	p<0.001	p<0.001	p=0.003	p=0.32	p<0.001	p<0.001		
16. Having children	0.01	-0.04	-0.12	-0.11	-0.12	-0.04	0.05	0.05	0.03	0.07	-0.02	0.11	0.44	0.03	-0.14	
	p=0.47	p=0.001	p<0.001	p<0.001	p<0.001	p=0,009	p<0.001	p<0.001	p=0.055	p<0.001	p=0.171	p<0.001	p<0.001	p=0.01	p<0.001	
17. Intention to stay in Germany	0.16	-0.17	0.13	0.15	0.13	0.11	0.01	0.09	-0.03	-0.08	0.01	-0.17	-0.06	-0.05	0.01	-0.06
	p<0.001	p<0.001	p<0.001	p<0.001	p<0.001	p<0.001	p=0,381	p<0.001	p=0,035	p<0.001	p=0,814	p<0.001	p<0.001	p<0.001	p=0.56	p<0.001
